# Fingolimod potentiates the antifungal activity of fluconazole against fluconazole-resistant *Candida auris*

**DOI:** 10.3389/fcimb.2025.1631780

**Published:** 2025-09-23

**Authors:** Ji-Hui Bae, Yong-Bin Eom

**Affiliations:** ^1^ Department of Biomedical Laboratory Science, College of Medical Sciences, Soonchunhyang University, Asan, Chungnam, Republic of Korea; ^2^ Department of Medical Sciences, Graduate School, Soonchunhyang University, Asan, Chungnam, Republic of Korea

**Keywords:** fingolimod, antifungal, anti-biofilm, synergistic effect, fluconazole-resistant *Candida auris*

## Abstract

*Candida auris* has emerged as a critical nosocomial pathogen that particularly affects immunocompromised and critically ill patients in intensive care units, and it is associated with high mortality. The robust biofilm-forming ability and inherent fluconazole resistance of *C. auris* pose considerable treatment challenges. Consequently, novel therapeutic strategies are urgently needed to treat fluconazole-resistant *C. auris* (FRCA). This study investigated the antifungal effect of the combination of fluconazole with fingolimod against FRCA. The antifungal activity of fingolimod and resistance of *C. auris* to fluconazole were assessed using a minimum inhibitory concentration assay, and their interaction was evaluated through a checkerboard synergy assay. The combination treatment effectively inhibited early biofilm formation and eradicated mature biofilms, as demonstrated by the biofilm inhibition concentration and biofilm eradication concentration assays, respectively. The XTT reduction assay revealed a marked reduction in the metabolic activity of *C. auris*, which was further corroborated by confocal laser-scanning microscopy. Quantitative polymerase chain reaction revealed the downregulation of key virulence-associated genes, including *ERG11* (azole resistance), *CDR1* (efflux pump), and *KRE6* (extracellular matrix). Collectively, these findings indicate that the combination of fingolimod and fluconazole inhibits biofilm formation, fungal metabolic activity, and virulence-related gene expression. This study suggests that fingolimod could serve as an adjuvant to improve the efficacy of fluconazole against FRCA.

## Introduction

Each year, approximately 1.9 million cases of invasive fungal infections are reported globally. Among these, invasive candidiasis accounts for approximately 70% of infections, and these infections are primarily attributable to *Candida* spp., which are major opportunistic fungal pathogens (Fang et al., 2023). *Candida auris* was first discovered in Japan in 2009, and it has subsequently emerged as a major invasive pathogen with a global mortality rate of 30%–72% (Pappas et al., 2018; De Gaetano et al., 2024). *C. auris* colonizes the skin, wounds, and catheter surfaces, leading to catheter-related infections and severe complications in immunocompromised individuals and patients in the intensive care unit (Jeffery-Smith et al., 2018; Arensman et al., 2020). Because of its multidrug resistance, high mortality rate, and outbreak potential, the World Health Organization has classified *C. auris* as a critical priority pathogen in its Fungal Priority Pathogens List, highlighting the need for global surveillance and action (Kim et al., 2024).

This clinical threat of *C. auris* is closely linked to its biofilm-forming ability, which is facilitated by its strong adherence to indwelling medical devices such as central venous catheters (Malinovská et al., 2023). Biofilms, structured microbial communities encased in an extracellular matrix, provide protection against antifungal agents and host immune responses (Estivill et al., 2011; Tuon et al., 2023). The high biofilm-forming capacity of *C. auris* is well established (Kean and Ramage, 2019). This characteristic contributes to its increased resistance by limiting the effectiveness of current antifungal therapies (Horton and Nett, 2020).

Antifungal resistance of *C. auris* is associated with multiple mechanisms, including mutations in azole resistance genes, efflux pump activity, and extracellular matrix (ECM) production (Kean et al., 2018). Azole-class drugs exert their effect by inhibiting essential enzymes involved in ergosterol biosynthesis, a key component of the fungal cell membrane, thereby impeding its formation. However, mutations in *ERG11* reduce the binding affinity of azole drugs, leading to resistance (Kelly et al., 1995).

In addition, efflux pumps play a critical role in expelling toxic compounds, including antifungals, from the cell (Chaabane et al., 2019). Overexpression of *CDR1*, which encodes an ABC-type efflux pump recognized as a major transporter in *Candida* species, increases the efflux of antifungals and contributes to multidrug resistance (Rybak et al., 2022). Meanwhile, the ECM is a complex polysaccharide structure that provides structural stability and cellular adhesion within the biofilm. Therefore, the ECM limits the penetration of antifungal agents and enhances biofilm persistence (Mitchell et al., 2016). The expression of *KRE6*, a gene involved in ECM production, confers additional resistance to osmotic stress and disinfectants commonly used in healthcare settings (Rybak et al., 2022). These mechanisms collectively reduce the efficacy of conventional treatments, highlighting the need for new therapeutic strategies in clinical management.

Fingolimod, a derivative of the immunosuppressive metabolite myriocin, is a Food and Drug Administration-approved treatment for multiple sclerosis (Aktas et al., 2010; Castro-Borrero et al., 2012). A previous study reported that fingolimod exhibits antibacterial effect (Jin et al., 2022). However, a few studies have investigated the antifungal activity of fingolimod against *C. auris*. Furthermore, its potential as an adjuvant to existing antifungal agents remains largely unexplored. Therefore, we evaluated the antifungal activity of fingolimod and its combined effects with fluconazole against fluconazole-resistant *C. auris* (FRCA).

## Materials and methods

### Organism, growth conditions, and reagents

Clinical isolates were obtained from the National Center Collection for Pathogens (Seoul, Korea) and the Korea Biobank Network (Daegu, Korea). All strains were subcultured on Sabouraud dextrose agar (Becton, Dickinson and Company, Franklin Lakes, NJ, USA) and then inoculated into Sabouraud dextrose broth (SDB; Becton, Dickinson, and Company) at 37°C. Fingolimod and fluconazole were purchased from TCI (Tokyo, Japan). Both compounds were dissolved in dimethyl sulfoxide (DMSO), and the final DMSO concentration did not exceed 2% in any assay.

### Minimum inhibitory concentration assay

MIC assays were performed to assess fluconazole resistance in clinical isolates and evaluate the antifungal efficacy of fingolimod. The assays were conducted using a broth microdilution method, with slight modifications based on the European Committee on Antimicrobial Susceptibility Testing (EUCAST) guidelines. Fungal suspensions (1 × 10^6^ CFU/mL) were treated with fingolimod, dispensed into 96-well plates (BD Falcon™, Becton, Dickenson and Company), and incubated at 37°C for 24 h. Absorbance was measured at 600 nm using a Multiskan™ GO Microplate Reader (Thermo Fisher Scientific, Waltham, MA, USA). The MIC was defined as the lowest concentration that inhibited ≥98% of fungal growth compared with the untreated control.

### Checkerboard synergy assay

The synergistic effect of fluconazole and fingolimod against FRCA was evaluated using a checkerboard assay with a 2-fold serial dilution method (Kim and Eom, 2021a). Fluconazole (16–256 µg/mL) and fingolimod (0.125–16 µg/mL) were distributed in ascending concentrations along the rows and columns of a 96-well plate to create combination matrices, with the highest concentration corresponding to their respective MICs. After incubation at 37°C for 24 h, absorbance was measured at 600 nm using a Multiskan™ GO Microplate Reader. The fractional inhibitory concentrations (FICs) of fluconazole and fingolimod were calculated using the following formulas: FIC of fluconazole = MIC of fluconazole in combination/MIC of fluconazole alone; and FIC of fingolimod = MIC of fingolimod in combination/MIC of fingolimod alone. The sum of the individual FICs was defined as the fractional inhibitory concentration index (FICI). Based on the FICI, the interactions were classified as synergistic (FICI ≤ 0.5), indifferent (0.5 < FICI ≤ 4.0), or antagonistic (FICI > 4.0).

### Biofilm inhibition concentration assay

The BIC assay was performed to evaluate the combined inhibitory effect of fluconazole and fingolimod on early biofilm formation by FRCA (Kim and Eom, 2021b). Fungal suspensions (1 × 10^6^ CFU/mL), treated with the drugs alone (16 µg/mL fluconazole, 2 µg/mL fingolimod) or in combination, were dispensed into 96-well plates and incubated at 37°C for 24 h. After incubation, the supernatant was removed, and each well was washed twice with phosphate-buffered saline (PBS). The plate was then dried in a 60°C oven for 1 h to fix the biofilm, followed by staining with 0.5% crystal violet for 7 min. The excess stain was removed by washing with distilled water, and the plate was further dried for 30 min. Subsequently, 30% acetic acid was added to each well, followed by incubation at room temperature for 20 min to solubilize the biofilm. Finally, absorbance was measured at 595 nm to assess biofilm inhibition.

### Biofilm eradication concentration assay

The BEC assay was conducted to evaluate the combined ability of fluconazole and fingolimod to eradicate preformed biofilms of FRCA (Kim et al., 2019). Untreated fungal suspensions (1 × 10^6^ CFU/mL) were dispensed into 96-well plates and incubated at 37°C for 24 h to permit biofilm formation. After incubation, the supernatant was removed, and each well was washed twice with PBS. The preformed biofilms were then treated with the drugs alone (16 µg/mL fluconazole, 2 µg/mL fingolimod) or in combination for 24 h. Biofilm staining was performed using the same protocol described for the BIC assay.

### XTT reduction assay

The metabolic activity of fungal cells within the biofilm was quantitatively assessed using the XTT reduction assay (Kim et al., 2020). Untreated fungal suspensions (1 × 10^6^ CFU/mL) were dispensed into 96-well plates and incubated at 37°C for 24 h. After incubation, the supernatant was removed, and each well was washed twice with PBS. The preformed biofilms were then treated with the drugs alone (16 µg/mL fluconazole, 2 µg/mL fingolimod) or in combination, followed by an additional 24-h incubation. Subsequently, the wells were washed twice with PBS, and 50 µL of the XTT reagent mixture were added to each well. The plates were incubated for 3 h, and absorbance was measured at 475 nm and 660 nm using a Multiskan™ GO Microplate Reader.

### Confocal laser-scanning microscopy

The metabolic activity within the biofilm was visually assessed using CLSM. Fungal suspensions (1 × 10^6^ CFU/mL) were dispensed into 24-well glass-bottom imaging plates and incubated at 37°C for 24 h to permit biofilm formation (Kim et al., 2022). Following incubation, the supernatant was removed, and the plate was washed twice with PBS. The preformed biofilms were then treated with either fingolimod (2 µg/mL) or fluconazole (16 µg/mL) alone or both drugs together for 24 h. Subsequently, the wells were washed twice with 0.85% NaCl, and the biofilms were stained using the LIVE/DEAD™ BacLight™ Bacterial Viability Kit (Thermo Fisher Scientific). Live cells were stained with SYTO9, and dead cells were stained with propidium iodide. Imaging was conducted at ×40 magnification, with fluorescence measured at 525 nm for SYTO9 and 640 nm for propidium iodide. The relative fluorescence intensity was quantified using ImageJ (RRID: SCR_003070) software.

### RNA isolation and quantitative polymerase chain reaction

For the transcriptomic analysis of FRCA, total RNA was extracted following treatment. Fungal suspensions (1 × 10^6^ CFU/mL) were inoculated into SDB and incubated at 37°C for 10 h. Subsequently, subinhibitory concentrations of the drugs in combination were administered, followed by incubation for 18 h. After incubation, the cultures were centrifuged at 10,000 rpm for 15 min at 4°C to collect the cell pellets. RNA extraction and purification were performed using the NucleoSpin RNA Mini Kit (Macherey-Nagel, Düren, Germany) according to the manufacturer’s instructions. The purity and concentration of the extracted RNA were assessed using a μDrop™ plate (Thermo Fisher Scientific).

The effects of treatment on the expression of virulence-related genes in FRCA were evaluated using qPCR. cDNA was synthesized using ReverTraAce qPCR RT Master Mix with gDNA Remover (TOYOBO, Osaka, Japan) following the manufacturer’s protocol. qPCR was conducted using TOPreal™ qPCR 2× PreMIX (Enzynomics, Daejeon, Korea) on a StepOnePlus™ Real-Time PCR System (Applied Biosystems, Foster City, CA, USA). *ACT1* was used as the endogenous control gene. The qPCR thermal cycling conditions included initial denaturation at 95°C for 10 min, followed by 40 cycles of denaturation at 95°C for 10 s, annealing at 60°C for 15 s, and extension at 72°C for 30 s. Melting curve analysis was performed at the end of the amplification under the following conditions: 95°C for 15 s, 58°C for 1 min, and 95°C for 15 s (Jin and Eom, 2025). Relative gene expression was normalized to *ACT1* expression and calculated using the 2^−ΔΔCT^ method. The primer sequences and annealing temperatures used in the analysis are listed in **Table 1**.

**Table 1:** Primers used for qPCR in this study.

Target gene	Primer sequence (5′-3′)	Annealing temp. (°C)	Reference
*ACT1*	F: TCCTCTCAGTCGTCCGCTAT	58	(Bhattacharya et al., 2019)
	R: CTTCATGGAAGATGGGGCTA		
*ERG11*	F: GTGCCCATCGTCTACAACCT	56	(Bhattacharya et al., 2019)
	R: TCTCTCTGCACAGCTCGAAA		
*CDR1*	F: GCCAGGTTTCTGGATTTTCA	55	(Bhattacharya et al., 2019)
	R: GGCCACAAGTTTGACCACTT		
*KRE6*	F: ATCACGATCGACATGGGCTC	55	(Short et al., 2019)
	R: TCAACGACAACGAAAACGGC		

### Statistical analysis

All analytical data are presented as the mean ± standard deviation, and statistical significance compared with the control group was evaluated using one-way ANOVA. Graphs were generated using GraphPad Prism version 10 (GraphPad Prism, Boston, MA, USA).

## Results

### MIC of clinical *C. auris* isolates

Fluconazole resistance and the antifungal activity of fingolimod were evaluated in clinical *C. auris* isolates (**Table 2**). Fingolimod exhibited strong antifungal activity, inhibiting fungal growth by more than 98% at concentrations of 4 and 16 µg/mL. The MICs of fluconazole ranged from 4 to 256 µg/mL, exhibiting considerable variability. According to the EUCAST guidelines, *C. auris* NCCP32683, *C. auris* NCCP32640, and *C. auris* KBN12P07096 were classified as fluconazole-resistant strains.

**Table 2:** MICs of fingolimod and fluconazole against clinically isolated *C. auris* strains.

Strains	Specimens	MIC (μg/mL)	
		Fingolimod	Fluconazole
*C. auris* NCCP32683	Blood	16	256
*C. auris* NCCP32640	Ear pus	16	256
*C. auris* NCCP32641	Blood	16	16
*C. auris* NCCP32684	Blood	16	16
*C. auris* NCCP32685	Blood	16	4
*C. auris* KBN12P06708	Ear pus	16	8
*C. auris* KBN12P07096	Ear pus	4	256

NCCP, National Culture Collection for Pathogens; KBN, Korea Biobank Network.

### Checkerboard assay of fluconazole and fingolimod

A checkerboard assay was conducted to investigate the combined effect of fluconazole and fingolimod against FRCA strains (**Table 3**). Synergy was defined as inhibition of fungal growth by ≥90% by combinations of subinhibitory concentrations. Fluconazole and fingolimod displayed synergy at concentrations of 16 µg/mL + 2 µg/mL for *C. auris* NCCP32640, 16 µg/mL + 4 µg/mL for *C. auris* NCCP32683, and 16 µg/mL + 1 µg/mL for *C. auris* KBN12P07096. The FICIs for these combinations were 0.1875, 0.3125, and 0.3125, respectively, including synergy. Based on these results, the biofilm assay was performed using *C. auris* NCCP32640, which had the lowest FICI.

**Table 3:** Synergistic effects of fingolimod and fluconazole against fluconazole-resistant *C. auris* strains.

Strains	MIC (μg/mL)			Synergistic effect	
	Fingolimod	Fluconazole	Fingolimod + fluconazole	FICI	Interpretation*
*C. auris* NCCP32640	16	256	2 + 16	0.1875	S
*C. auris* NCCP32683	16	256	4 + 16	0.3125	S
*C. auris* KBN12P07096	4	256	1 + 16	0.3125	S

*S, synergism; I, indifference.

### The inhibitory effect of fluconazole and fingolimod on early biofilm formation by FRCA

**Figure 1:**
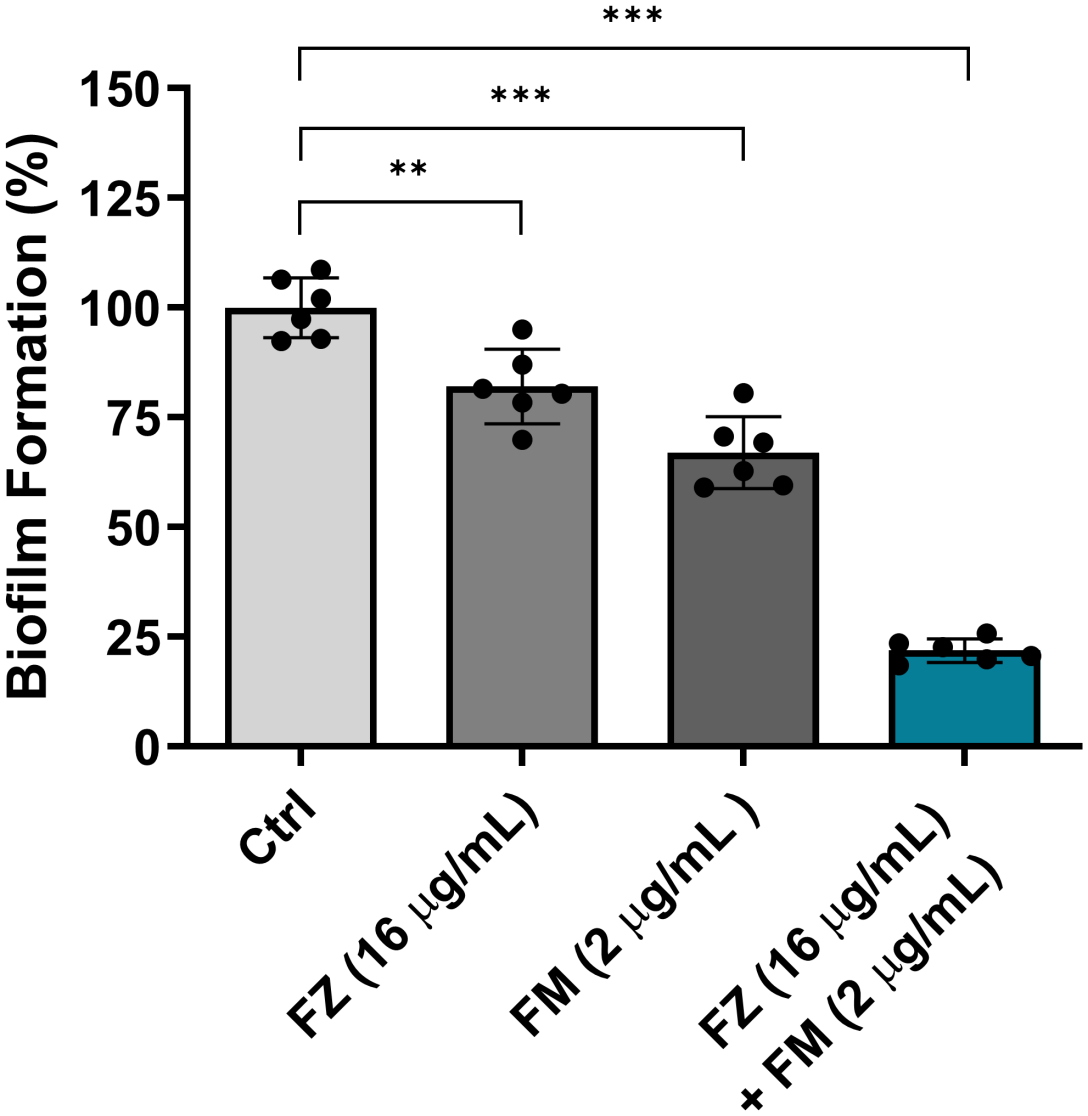
Inhibitory effect of fluconazole (FZ) and fingolimod (FM) on biofilm formation by *C. auris* NCCP32640. Biofilm formation was evaluated following treatment with FM and/or FZ. The biofilms were stained with 0.5% crystal violet, and absorbance was measured at 595 nm. Positive control (Ctrl) contains 2% DMSO. Statistical significance was assessed relative to the untreated control, with significant differences indicated by asterisks (**p ≤ 0.01, ***p ≤ 0.001). Data are presented as the mean ± standard deviation (SD), and error bars represent the SD.

The inhibitory effect of the synergistic combination of fluconazole and fingolimod on early biofilm formation was evaluated using *C. auris* NCCP32640 (**Figure 1**). Individual treatments with fluconazole and fingolimod were administered at the concentrations used in the synergistic combination. When applied alone, fluconazole and fingolimod inhibited biofilm formation by approximately 17% and 33%, respectively. By contrast, their combination significantly inhibited early biofilm formation by up to 78%.

### The eradication effect of fluconazole and fingolimod on the mature biofilms of FRCA

**Figure 2:**
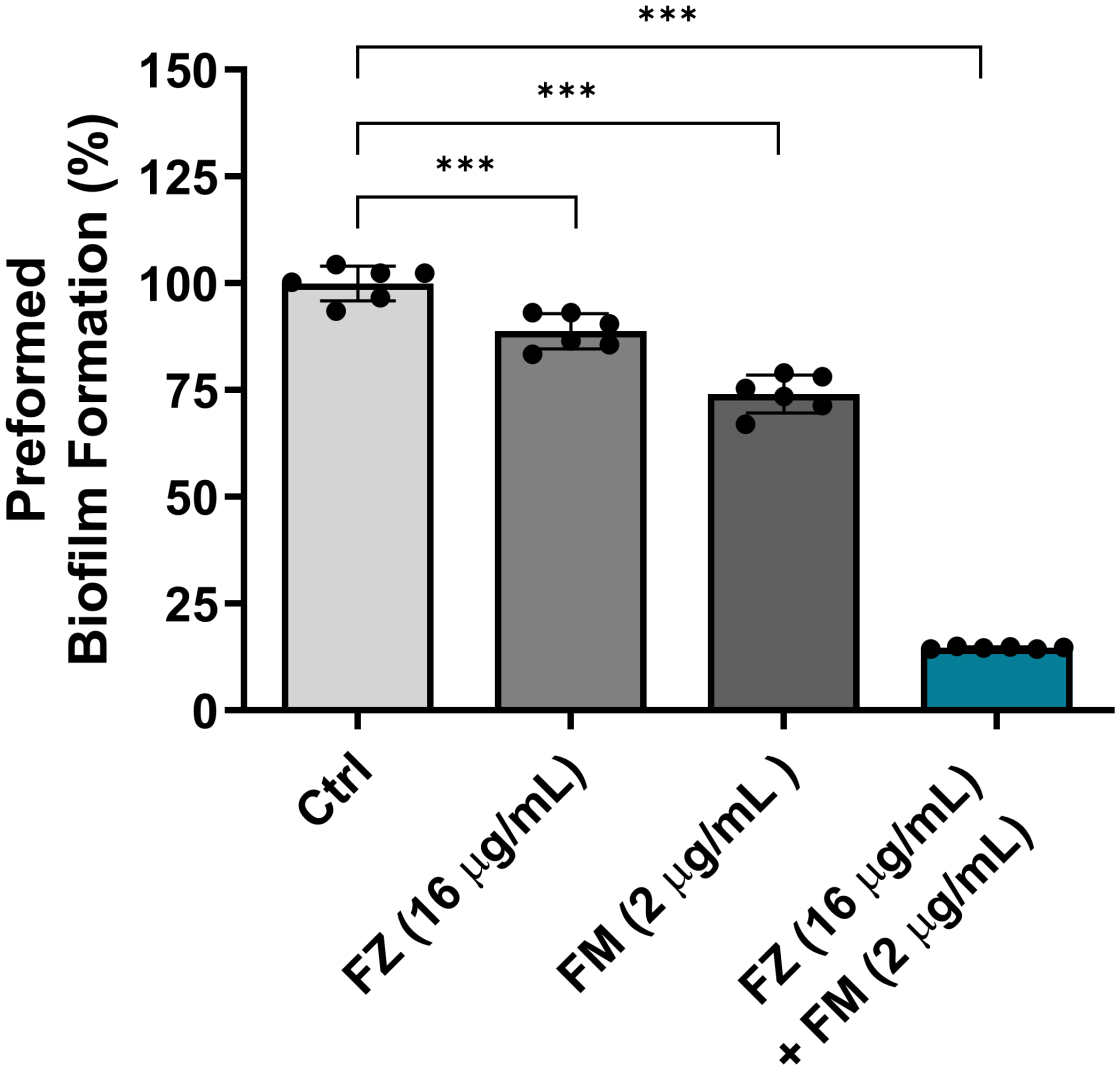
Eradication effect of fluconazole (FZ) and fingolimod (FM) on preformed biofilms of *C. auris* NCCP32640. The fungal suspensions were incubated for 24 h to allow biofilm formation, washed with PBS and treated with FZ and/or FM. The remaining biofilms were stained with 0.5% crystal violet, and absorbance was measured at 595 nm. Positive control (Ctrl) contains 2% DMSO. Statistical significance was assessed relative to the untreated control, with significant differences indicated by asterisks (***p ≤ 0.001). Data are presented as the mean ± standard deviation (SD), and error bars represent the SD.

The efficacy of the synergistic combination of fluconazole and fingolimod in eradicating the preformed biofilms of *C. auris* NCCP32640 was evaluated (**Figure 2**). Individual treatments with fluconazole and fingolimod were applied at concentrations used in their synergistic combination. When applied separately, fluconazole and fingolimod achieved inhibition rates of approximately 11% and 26%, respectively. However, combination treatment eradicated approximately 85% of the preformed biofilms.

### The effect of the synergistic combination on the metabolic activity of FRCA

**Figure 3:**
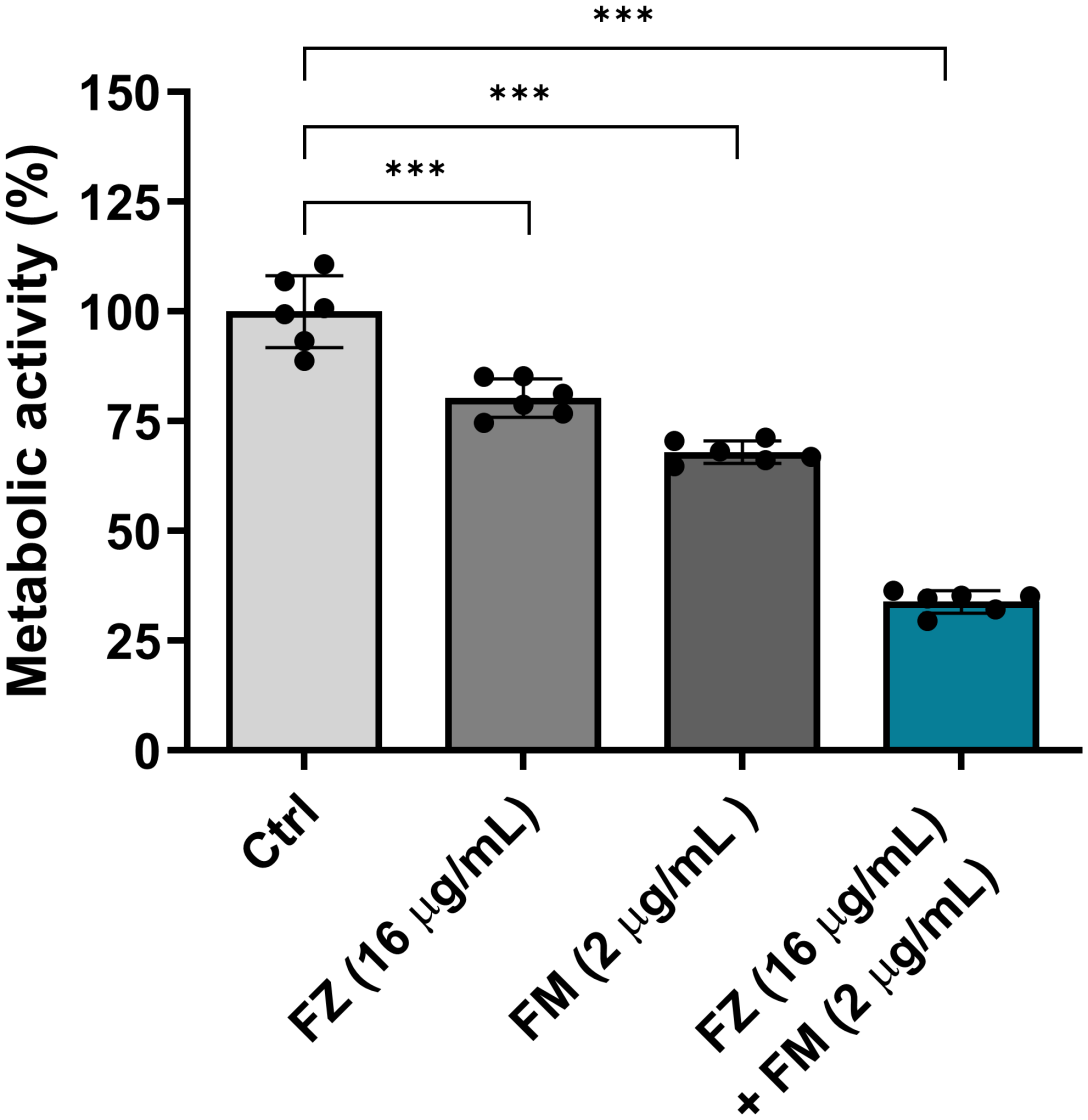
Antifungal effect of fluconazole (FZ) and fingolimod (FM) on the metabolic activity of *C. auris* NCCP32640 in biofilms. Metabolic activity was quantitatively analyzed by measuring the color changes resulting from the reduction of XTT, which reflects cell viability. Absorbance was measured at 475 and 660 nm, and values were calculated using the following formula: A_475_ nm (test) − A_475_ nm (blank) − A_660_ nm (test). A decrease in absorbance indicates reduced metabolic activity in *C. auris* and an enhanced antifungal effect. Positive control (Ctrl) contains 2% DMSO. Statistical significance was assessed relative to the untreated control, with significant differences indicated by asterisks (***p ≤ 0.001). Data are presented as the mean ± standard deviation (SD), and error bars represent the SD.

We confirmed that fluconazole and fingolimod inhibited the metabolic activity of fungal cells within the biofilms (**Figure 3**). When used individually, fluconazole and fingolimod inhibited the metabolic activity of FRCA by approximately 20% and 32%, respectively. In comparison, when applied in combination, a significant reduction in metabolic activity of approximately 66% was observed.

### Visualization of the metabolic activity of fungal cells within biofilms

**Figure 4:**
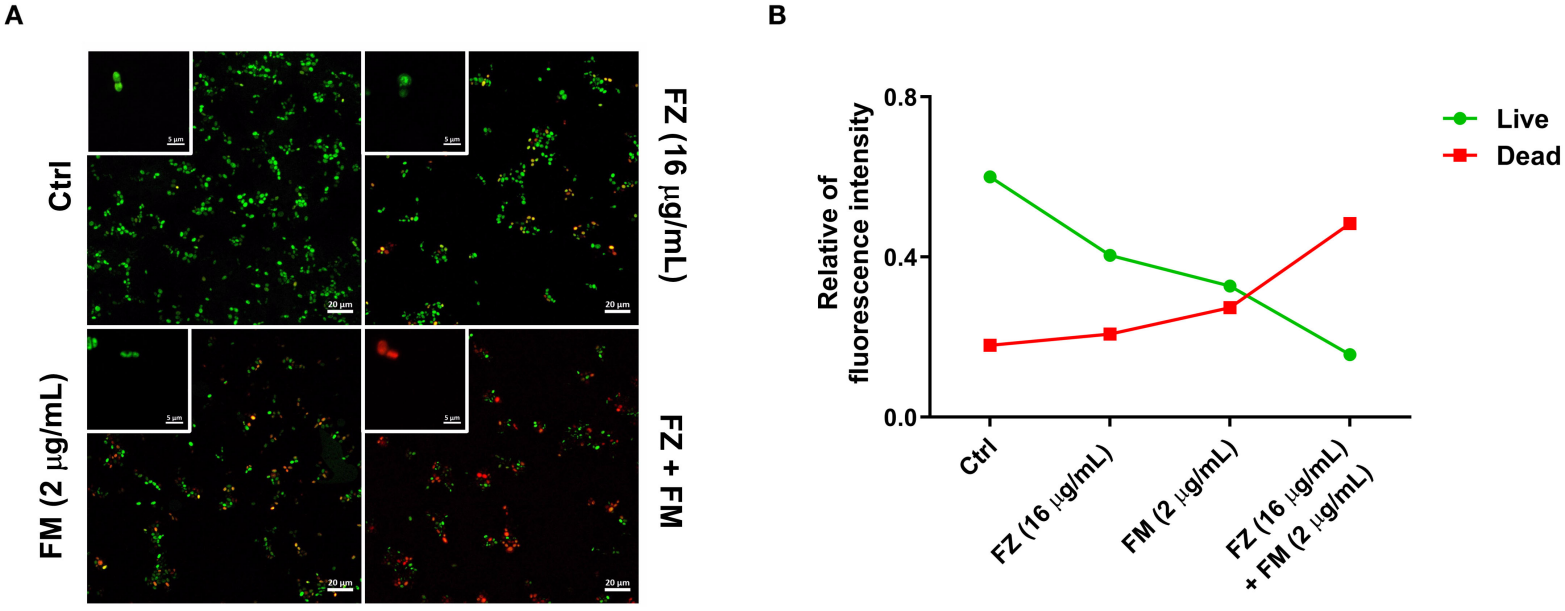
**(A)** CLSM images presenting cell viability within *C*. *auris* NCCP32640 biofilms after treatment with fluconazole (FZ) and fingolimod (FM). Live cells (green) and dead cells (red) were distinguished using LIVE/DEAD staining with SYTO9 and propidium iodide, respectively. Images were captured at ×40 and ×1000 magnification (scale bar, 20 µm). **(B)** Quantitative analysis of the relative fluorescence intensity comparing the ratio of live cells to dead cells based on the CLSM images.

As presented in **Figure 4A**, strong green fluorescence, indicating living cells, was observed in the control group, whereas an increase in red fluorescence, which is associated with dead cells, was detected for the synergistic combination. Fluorescence analysis (**Figure 4B**) further confirmed that the combination treatment increased the number of dead cells and decreased the number of live cells compared with the findings in the control group.

### Expression of virulence-related genes in FRCA

**Figure 5:**
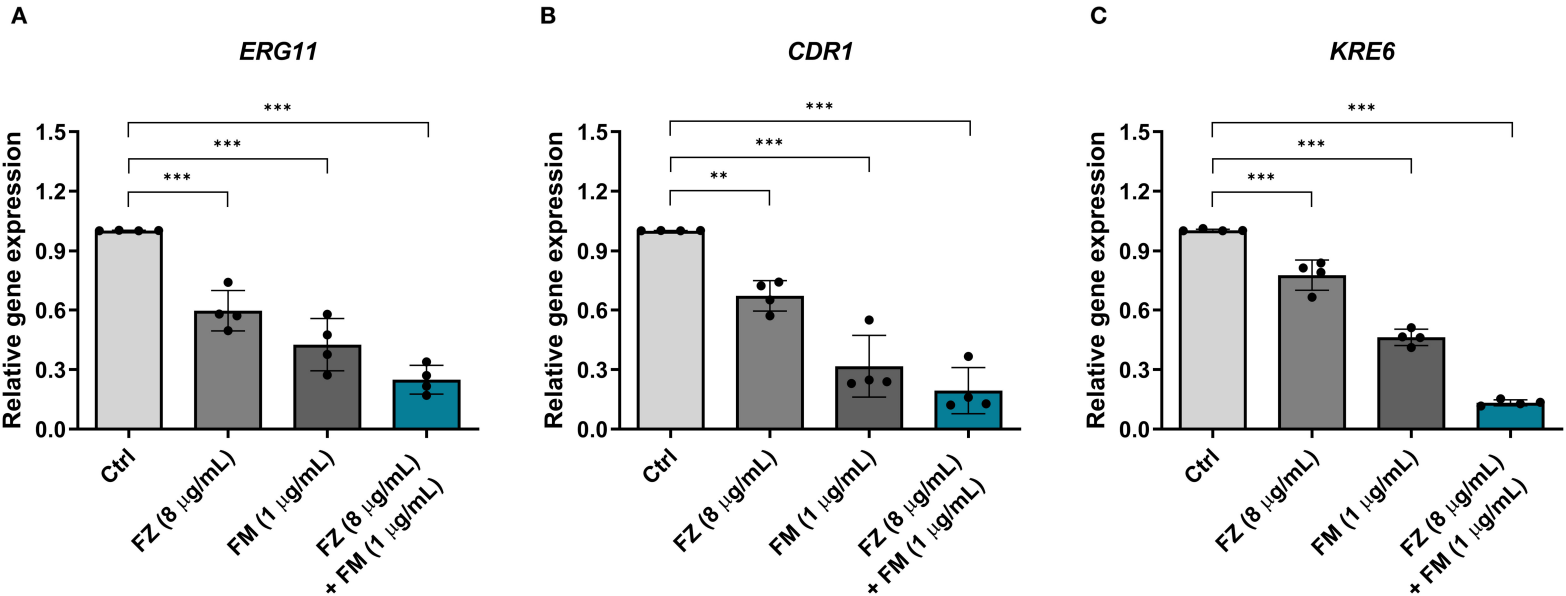
Relative expression of virulence-related genes in *C*. *auris* NCCP32640 treated with subinhibitory concentrations of fluconazole (FZ) and fingolimod (FM). The relative expression of **(A)** the azole resistance gene *ERG11*, **(B)** efflux pump gene *CDR1*, and **(C)** ECM-associated gene *KRE6* was analyzed. The expression of target genes was normalized to the expression of *ACT1* as an internal control. Positive control (Ctrl) contains 2% DMSO. Asterisks (*) indicate statistically significant differences compared with the control (**p < 0.01, ***p < 0.001). Data are presented as the mean ± standard deviation (SD), and error bars represent the SD.

qPCR was conducted to examine the changes in the expression of virulence-related genes in *C. auris* NCCP32640 after treatment with fluconazole and fingolimod (**Figure 5**). The synergistic combination considerably reduced the expression of the azole resistance gene *ERG11* by approximately 75% compared to the control group. Additionally, the expression of the efflux pump gene *CDR1* and ECM-associated gene *KRE6* was suppressed by approximately 81% and 87%, respectively. These results suggest that the combination of fluconazole and fingolimod effectively downregulates key virulence-related genes.

## Discussion

*C. auris* is a major invasive fungal pathogen responsible for invasive candidiasis because of its strong biofilm-forming ability, leading high mortality rates (Lockhart, 2019; Du et al., 2020). This issue is particularly critical in healthcare settings, in which the limitations of current treatment options have become increasingly apparent, underscoring the urgent need for novel therapeutic strategies (Cristina et al., 2023). Fingolimod, a clinically approved drug for the treatment of multiple sclerosis, has a well-established safety profile. Repurposing already approved therapeutics could offer a more efficient approach than developing entirely new drugs (Gilbert-Girard et al., 2020).

Fingolimod has been reported to exhibit antibacterial and antifungal activities, with effective concentrations of 3.125 µg/mL against *Staphylococcus aureus*, 4 μg/mL against *Clostridium perfringens*, and 0.25 mg/mL (MIC_99_) against *C. albicans* (Rumah et al., 2017; Najarzadegan et al., 2022; Shang et al., 2024). However, antifungal research related to *C. auris* remains limited. Previous studies have demonstrated that fingolimod did not cause cytotoxicity in the normal human colon mucosal epithelial cell line (NCM460) at concentrations below 11 mg/L (Wei et al., 2021). In this study, fingolimod exhibited antifungal activity at concentrations of 4 and 16 μg/mL against clinical *C. auris* isolates obtained from patients (**Table 2**). These findings suggest that fingolimod exhibits not only antibacterial effects but also antifungal effects, even at concentrations similar to or significantly lower than those used in previous studies.

Numerous studies have reported that various drugs can serve as adjuvants to fluconazole. In one study, the MIC of fluconazole for *C. albicans* remained at 0.5 μg/mL even when combined with the calcineurin inhibitor cyclosporine. However, the researchers introduced a new concept, “MIC-0,” to show a strong synergistic effect with the combinations of fluconazole (0.5 μg/mL) and cyclosporine (0.625 μg/mL) (Marchetti et al., 2000). Another study reported that the combination of fluconazole (32 μg/mL) and cyclosporine A (75 μg/mL) significantly inhibited *C. albicans* biofilm growth and suppressed the expression of resistance-associated genes (*CDR1*, *MDR1*, *ERG11*) (Jia et al., 2016). Similarly, the combination of the fluconazole (1 μg/mL) and Hsp90 inhibitor ganetespib (0.5 μg/mL) exhibited remarkable antifungal activity against azole-resistant *C. albicans*. At higher concentrations (8 μg/mL each), this combination further downregulated the expression of key resistance genes (*CDR1*, *CDR2*, *MDR1*, *ERG11*) (Yuan et al., 2021).

Based on this, we investigated whether fingolimod and fluconazole can exert synergistic effects. A synergistic effect was observed with the combination of 1–4 μg/mL fingolimod and 16 μg/mL fluconazole against FRCA (**Table 3**). In a previous study, a synergistic effect against *C. albicans* was reported for fingolimod combined with amphotericin B, resulting in approximately 80% growth inhibition (Wei et al., 2021). By contrast, our study demonstrated a more potent inhibitory effect, with *C. auris* growth reduced by more than 90% at concentrations 2–4-fold lower than the individual MICs of the two drugs. Considering that fluconazole is a first-line treatment for fungal infections, the combined use of fluconazole and fingolimod might have significant therapeutic implications in treating drug-resistant *C. auris* infections.

The robust biofilm formation of *C. auris* is associated with over 80% resistance to fluconazole, highlighting biofilms as an important therapeutic target (Sati et al., 2022). Accordingly, we assessed the antibiofilm effects of the synergistic combination using BIC and BEC assays. Previous studies reported that 50 μM fingolimod inhibits biofilm formation and reduces biofilm thickness in *Pseudomonas aeruginosa* (Niazy et al., 2024). In this study, the combination therapy suppressed biofilm formation by 78% and effectively eradicated mature biofilms by 85%. (**Figures 1**, **2**). These findings support the notion that fingolimod has the potential to disrupt biofilms in both bacterial and fungal pathogens.

Crystal violet, used in BIC/BEC assays, both viable and non-viable cells as well as the ECM. Although this enables quantification of the total biofilm biomass, it does not accurately reflect cell viability (Xu et al., 2016). Therefore, we employed an XTT reduction assay to specifically assess the metabolic activity of the living cells within the biofilm. The results revealed a significant reduction in metabolic activity following treatment with the synergistic combination, which was further supported by CLSM (**Figures 3**, **4**). These findings indicate a fungicidal effect on FRCA with combination treatment as opposed to perhaps fungistasis in the singular treatments.

The antifungal resistance of *C. auris* is attributed to the interplay of multiple resistance mechanisms. Accordingly, we conducted qPCR analysis to assess the effect of combination therapy on the expression of key virulence-associated genes implicated in resistance, namely *ERG11*, *CDR1*, and *KRE6* (**Figure 5**). The combination treatment significantly decreased the expression of all three genes, with the most pronounced reduction observed for *KRE6*. These findings suggest that the synergistic combination disrupts both structural and functional resistance mechanisms by reducing the expression of resistance-related genes.

However, as this study was conducted *in vitro*, it does not fully reflect the complex architecture of the biofilms formed *in vivo*. Therefore, additional *in vivo* studies are required to validate the clinical applicability of this combination therapy, its long-term efficacy within the host, and the potential for resistance development. This study did not elucidate the molecular pathways underlying fingolimod’s antifungal synergy or the mechanistic modeling by which it enhances fluconazole efficacy, underscoring the need for further investigation, including evaluation of its synergistic activity with other antifungals such as amphotericin B, particularly against the highly resistant *C. auris*.

## Conclusion

This study provides experimental evidence supporting the synergistic effect of fingolimod and fluconazole against FRCA. We demonstrated that the combination therapy exhibits antibiofilm effects, inhibits fungal metabolic activity, and decreases virulence-related gene expression. These findings suggest a promising approach for developing therapeutic strategies to address the limitations of current antifungal treatments.

## Data Availability

The data that support the findings of this study are available from the corresponding author upon reasonable request. Requests to access the datasets should be directed to Y-BE, omnibin@sch.ac.kr.
